# Pyrimidine-containing natural products: occurrences and biological activities

**DOI:** 10.1007/s13659-025-00583-y

**Published:** 2026-02-04

**Authors:** Jian-Neng Yao, Yongjie Zhu, He-Ping Chen, Yihua Chen

**Affiliations:** 1 School of Pharmaceutical Sciences and Yunnan Key Laboratory of Pharmacology for Natural Products, Kunming Medical University, Kunming, 650500 Yunnan China; 2 Yunnan College of Modern Biomedical Industry, Kunming Medical University, Kunming, 650500 Yunnan China; 3 School of Pharmaceutical Sciences, South-Central Minzu University, Wuhan, 430074 China

**Keywords:** Pyrimidine, Pyrimidine-containing natural products, Biological activities, Occurrences, Isolation and identification

## Abstract

Nitrogen-containing core structure pyrimidine has long been regarded as one of the privileged scaffolds for novel drug development. Natural products embedded with pyrimidine motifs are distinguished by their exceptional scaffold diversity and vast structural complexity, which endow them with versatile biological activities, including anticancer, antiviral, antifungal and anti-inflammatory activities. This review is dedicated to surveying a series of structurally distinctive naturally occurring compounds characterized by the presence of an aromatic heterocyclic pyrimidine moiety covering from 2004 to early 2025. Multiple key aspects of these 156 pyrimidine-containing compounds, including natural sources, features of chemical structure, biological activities, as well as biosynthetic studies are summarized. The review emphasizes the enduring potential of natural products, highlighting their inherent capacity for medication and optimization to fuel the pipeline of lead compound targeting major disease and providing new window for overview of these pyrimidine-containing natural products. Overall, this review aims to unlocking the potential of these isolates. It thus offers a fresh reference for the exploitation of pyrimidine-containing natural products, with the ultimate goal of realizing their therapeutic potential.

**Graphical Abstract:**

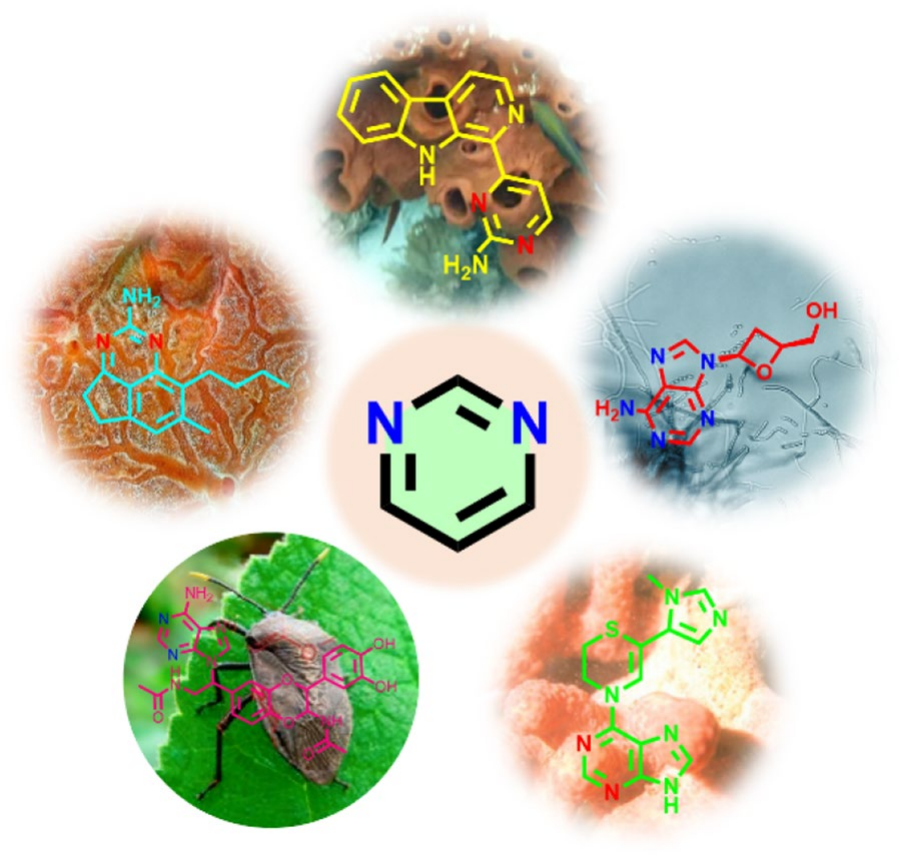

## Introduction

Natural products have consistently played an integral role in drug discovery and development, profoundly safeguarding human health through a multitude of therapeutic applications [1–3]. Irrefutably, a significant proportion of these natural occurrences have been used directly as active pharmaceutical ingredients or serve as chemical templates for further structural modification into new chemical entities. Moreover, natural products feature rich sp^3^-hybridzed carbons and novel skeletons, which extremely expand molecular diversity and enrich chemical space. These merits contribute to leverage natural product-based drug discovery spanning various medical treatments for diseases, including cancer, cardiovascular diseases, infectious diseases and central nervous system disorders. Additionally, natural products represent a valuable source of chemical probes for chemoproteomic studies designed to target identification and elucidating biological mechanisms [4, 5].

**Fig. 1:**
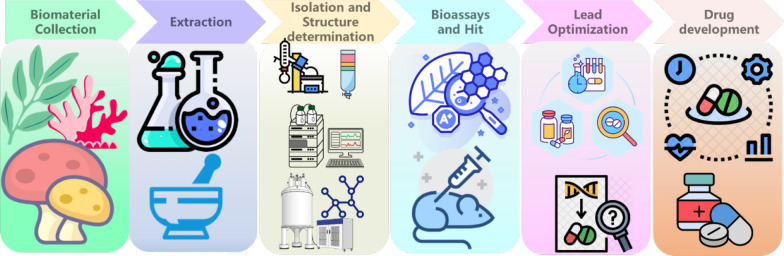
Key stages in the drug development pipeline derived from natural products

The discovery and development of therapeutics derived from natural products constitutes a multifaceted process involving multiple critical stages, each playing an indispensable role in translating the potential of isolated compounds into viable clinical drugs (Fig. 1).

**Fig. 2:**
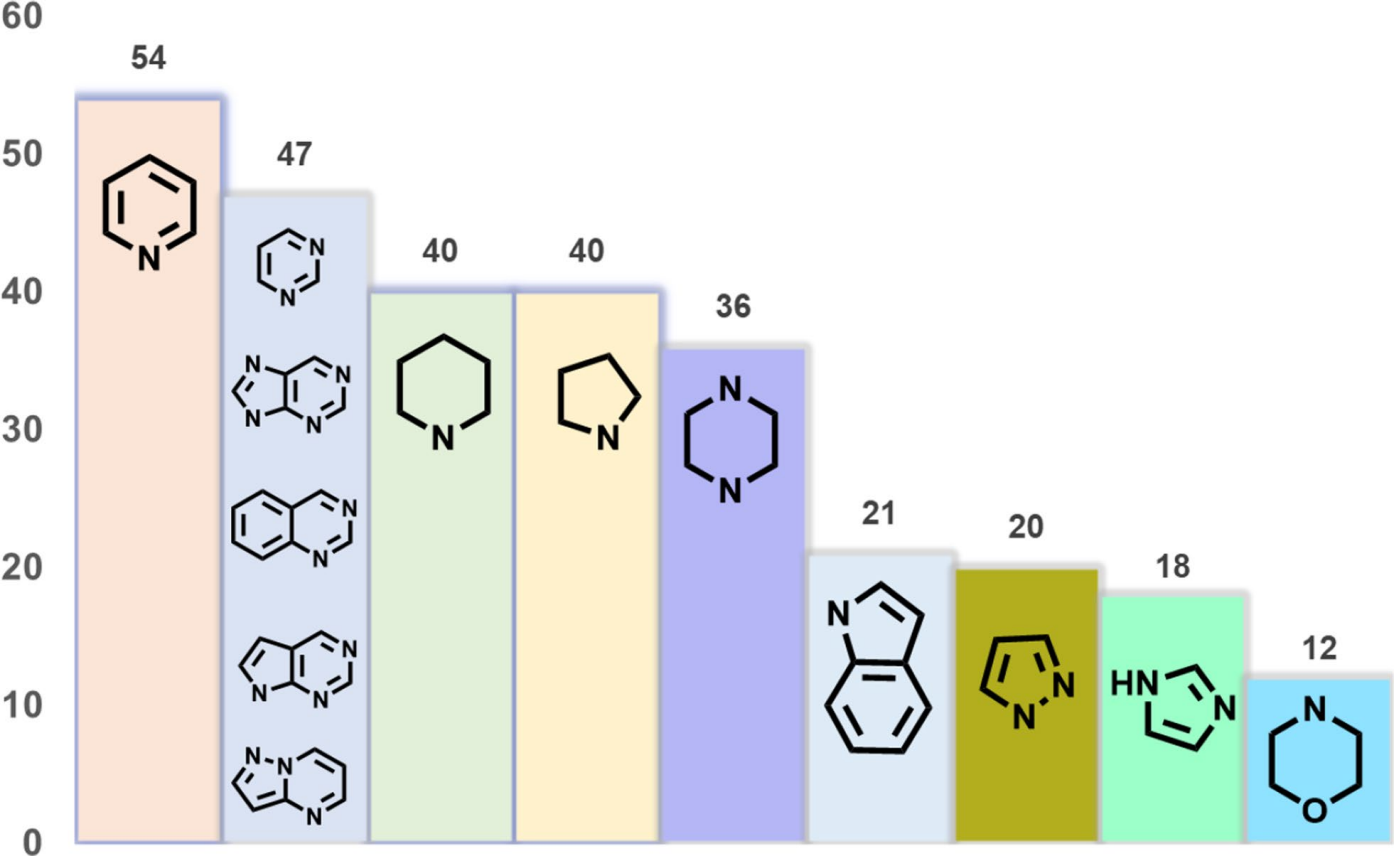
Top 10 nitrogen heterocycles in U.S. FDA approved drugs (January 2013–December 2023)

**Fig. 3:**
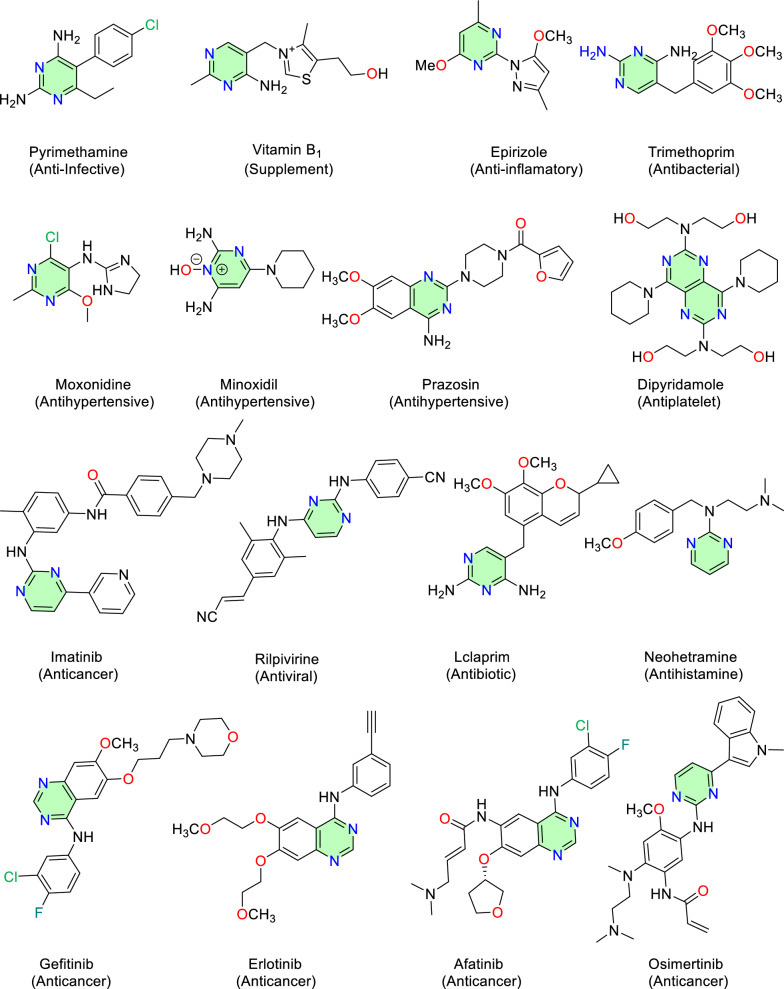
Chemical structures of marketed drugs containing the pyrimidine scaffold

Nitrogen heterocycles are widely distributed among approved drugs [6]. Pyrimidine-based heterocycles, for example, rank as the second most common ring systems in marketed pharmaceuticals (Fig. 2). Pyrimidine and its derivatives have been recognized as privileged scaffolds for drug development in recent years [7–11]. Several approved small-molecule drugs have showcased that pyrimidine ring system is essential to potent biological activities (Fig. 3), including anti-infective, antibacterial, antihypertensive, antiviral, antiplatelet and anticancer effects. Novel pyrimidine-based drug candidates have lured considerable attention, presumably due to its inherent capacity to enhance the binding affinity via hydrogen bond and/or to function as bioisosteres of the phenyl ring and other *π*-conjugated scaffolds in drug design [12]. Despite the enormous value of this pyrimidine core, comprehensive reviews on natural products that contain substituted or ring-fused pyrimidine ring systems remain relatively scarce compared with those on small-molecule compounds driven by synthetic approaches [13, 14].

Pyrimidine is an aromatic six-membered heterocyclic compound that consists of four carbon atoms and two nitrogen atoms located at positions 1 and 3. The name “pyrimidine” was first coined by the organic chemist Pinner in the early 1880s. Pinner also pointed out the structural similarity between pyrimidine and other aromatic compounds such as benzene and pyridine, underscoring the importance of its aromatization [15, 16]. It’s noteworthy that pyrimidine-containing natural products and their synthetic analogues continue to serve as irreplaceable engines for therapeutic innovation and aspiration, consistently affording structurally bioactive architectures, including meridianins A-H [17], cordycepin [18] and aplithianine A [19, 20]. Driven by long-standing interest in small-molecule compounds featuring the pyrimidine motif [21–26], this review offers a comprehensive structural profile of pyrimidine-containing natural products, along with an overview of their biological activities, covering the literature from 2004 to early 2025. Furthermore, this review mainly deals with natural products bearing aromatic heterocyclic ring system pyrimidine, while those natural products merging with nonaromatic pyrimidine derivatives, such as 2-pyrimidone, 4-pyrimidone, barbituric acid or three types of nucleobases (cytosine, thymine and uracil) are excluded.

In this review, 156 naturally occurring compounds containing pyrimidine-based motif have been reported, demonstrating a significant variety of chemical structures. The compounds in this review are categorized into three classes, including those with non-glycosylated pyrimidine-containing natural products, glycosylated pyrimidine derivatives and purine-containing natural products.

Notably, two seminal reviews compiled by Lagojia and Rosemeyer respectively have disclosed that the pyrimidine-containing natural products reported before 2004 [27, 28]. However, several pyrimidine-containing structures were unintentionally omitted in their reviews. Given the structural diversity of these natural products discussed and the significance of the topics highlighted, the missing molecules in previous reviews are included in this review.

## Non-glycosylated pyrimidine-containing natural products

### Non-fused pyrimidine-containing alkaloids

Pyrimidine-containing alkaloids represent a class of natural products characterized by the presence of one or more non-fused substituents on the pyrimidine scaffold. Common examples of such peripheral modifications include indole rings and benzyl groups.

**Fig. 4:**
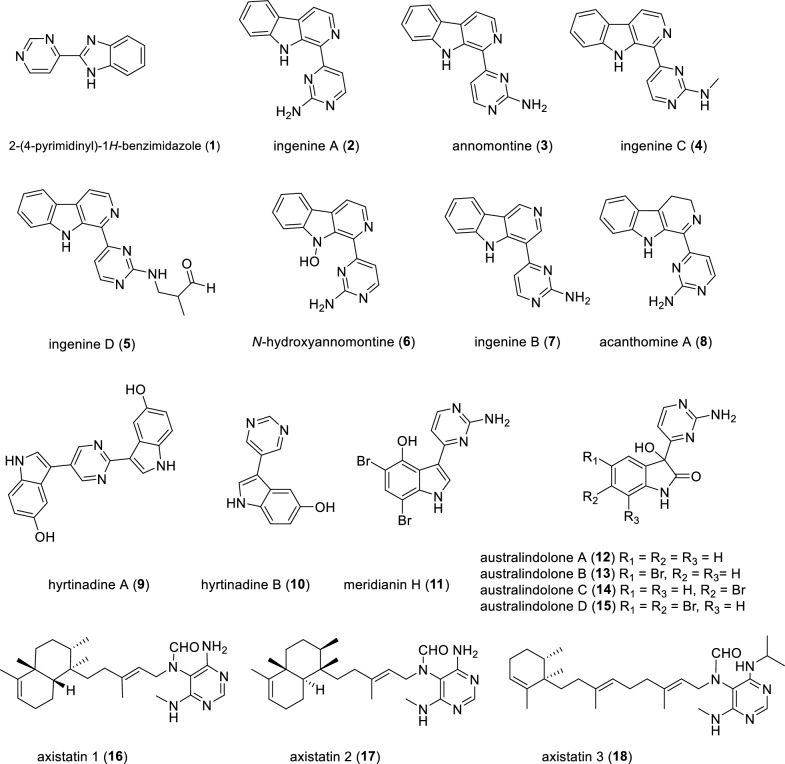
Chemical structures of compounds **1**–**18**

A monosubstituted pyrimidine derivative, 2-(4-pyrimidinyl)-1*H*-benzimidazole (**1**), was recently isolated from cultured mycelia of *Ophiocordyceps sinensis*, which is a conspicuous traditional medicine (Fig. 4). Its chemical structure was established on the basis of mass spectroscopic data analysis [29]. Previous literature indicates that compound **1** was first obtained via chemical synthesis as early as 1960 [30]. Compounds **2–5** are a new class of alkaloids featuring a pyrimidine-*β*-carboline moiety in their chemical structures. Ingenine A (**2**), annomontine (**3**) and ingenines C and D (**4** and **5**) were isolated from the Indonesian sponge *Acanthostrongylophora ingens* by Ibrahim and co-workers [31, 32]*.* Interestingly, the co-isolated alkaloids **2** and **3** showed significant discrepancies in their NMR chemical shifts around the pyrimidine motif and in their optical rotation (OR) values with specific rotations of $[\alpha]_{\mathrm{D}}^{25}$ + 16 for **2** and $[\alpha]_{\mathrm{D}}^{25}$ -34 for **3**. These observations suggest that compounds **2** and **3** may constitute a pair of atropisomers. However, the absolute configurations of both compounds (**2** and **3**) remain unresolved. Compound **3** exhibited pronounced cytotoxicity against the murine lymphoma L5178Y cancer cell line with ED_50_ (median effective dose) value of 7.8 μg·mL^−1^. Compounds **4** and **5** showed moderate inhibitory activity against MCF-7 and HCT-116 cell lines with IC_50_ (half maximal inhibitory concentration) values of 4.33 and 6.05 for **4**, and 2.90 and 3.35 μM for **5**, respectively. A novel antileishmanial pyrimidine-*β*-carboline alkaloid, *N*-hydroxyannomontine (**6**), was isolated from the bark of *Annona foetida* via bioassay-guided fractionation [33]. Compound **6** plays a key role in *Annona* genus chemotaxonomy. From the Indonesian sponge *Acanthostrongylophora ingens*, ingenine B (**7**), which features a pyrimidine-*γ*-carboline motif, was isolated and demonstrated cytotoxicity against lymphoma L5178Y cancer cell line with ED_50_ value of 9.1 μg·mL^−1^ [34]. Another metabolite, acanthomine A (**8**), also obtained from the sponge *A. ingens*, represents a new type of pyrimidine-dihyro-*β*-carboline alkaloid [34]. Furthermore, X-ray crystallographic analysis suggested that acanthomine A (**8**) seems to occur naturally as a racemate [35]. Hyrtinadine A (**9**), a bis-indole alkaloid with a *para*-substituted pyrimidine moiety, was isolated from the sponge *Hyrtios* sp. Compound **9** showed cytotoxicity against murine leukemia L1210 and human epidermoid carcinoma KB cell lines with IC_50_ values of 1 μg·mL^−1^ and 3 μg·mL^−1^, respectively [36, 37]. From the sponge *Scalarispongia* sp., an indole-pyrimidine alkaloid hyrtinadine B (**10**) was isolated and further evaluated its cytotoxicity against the human leukemia cell K562 cell line. Compound **10** showed negligible cytotoxicity [38].

The marine natural products meridianins A-G, characterized by indole-pyrimidine ring systems, represent a new family of protein kinase inhibitors [39, 40]. These alkaloids were purified from the South Atlantic Ascidian *Aplidium meridianum*. Since their discovery, continuous efforts have been devoted to exploring their biological activities in-depth and developing efficient chemical synthesis routes [41–43]. Recently, a new member of this family, meridianin H (**11**), along with four structurally related derivatives coupled with 2-aminopyrimidine ring australindolones A–D (**12–15)** were isolated from the deep water Antarctic tunicate *Synoicum* sp. [44].

Axistatins 1**–**3 (**16–18**) with pyrimidine diterpene scaffolds were identified from the Republic of Palau marine sponge *Agelas axifera*. Compounds **16–18** demonstrated moderate inhibitory activity against cancer cell growth and also exhibited antimicrobial activity [45].

### Fused pyrimidine alkaloids

**Fig. 5:**
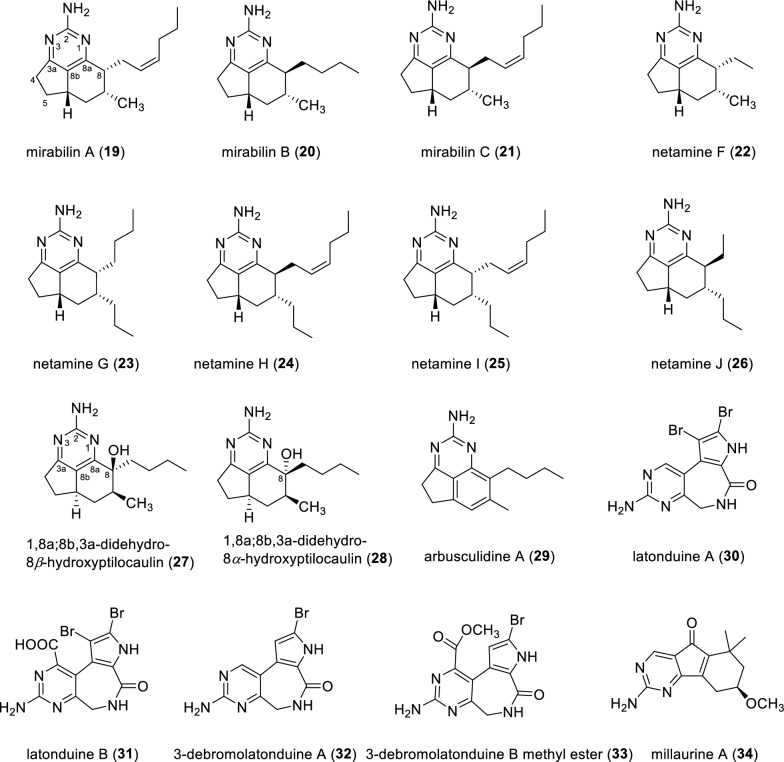
Chemical structures of compounds **19**–**34**

Fused pyrimidine alkaloids are characterized by the presence of two or more cyclic rings in the molecules (Fig. 5). Crucially, at least one of these rings is fused directly to the pyrimidine ring, forming new fused-ring frameworks. A total of 21 such compounds have been identified. Marine-derived mirabilins A–C (**19–21**) were isolated from the sponge *Arenochalina mirabilis*, collected from the Great Australian Bight [46, 47]. Netamines F–J (**22–26**) coupled with aromatic pyrimidine moieties were identified from the marine sponge *Biemna laboutei*. Interestingly, hydrogenation of netamine H over Pd/C with H_2_ afforded natamine A, suggesting that the relative configuration of the side chains embedded in the molecules is indeed identical. Biological evaluation results indicate that netamine K and mirabilin A exhibited antimalarial activity with IC_50_ values of 2.4 and 20.7 μM, respectively [48]. Two tricyclic alkaloids, 1,8a;8b,3a-didehydro-8*β*-hydroxyptilocaulin (**27**), and 1,8a;8b,3a-didehydro-8*α*-hydroxyptilocaulin (**28**) were uncovered from the marine sponge *Monanchora unguifera.* X-ray crystallographic analysis of co-crystallized compounds **27** and **28** confirmed these two compounds as a pair of C-8 epimers [49]. Co-existing compounds **27** and **28** showed antimalarial activity against the parasite *Plasmodium falciparum*, with an IC_50_ value of 3.8 μg·mL^−1^. Arbusculidine A (**29**) represents the first compound in the netamine family to feature a benzyl group, whose chemical structure is closely related to the family of tricyclic pyrimidine alkaloids [50]. From a broader perspective, compound **29** also belongs to the distinctive quinazoline skeleton.

Chemical investigation of the Indonesian marine sponge *Stylissa carteri* led to the isolation of two aminopyrimidine-containing alkaloids latonduines A and B (**30** and **31**). These two alkaloids with unprecedented tricyclic scaffolds were established by analysis of HRMS and NMR data and further confirmed by the total synthesis of compound **30**. Biosynthetically, amino acid ornithine likely serves as the biosynthetic precursor to the aminopyrimidine moiety of latonduines A and B. Both compounds **30** and **31** were evaluated for their in vitro cytotoxicities against a panel of human cancer cell lines and for inhibitory activities against a panel of protein kinases. Unfortunately, they were found to be inactive in these bioassays [51]. Two pyrrole-pyrimidine alkaloids, 3-debromolatonduine A (**32**) and 3-debromolatonduine B methyl ester (**33**), were identified from the marine sponge *Stylissa* sp. [52]. Millaurine A (**34**), a pyrimidine-containing alkaloid with 6/5/6 tricyclic skeleton, was isolated from the seeds of *Millettia laurentii* [53].

**Fig. 6:**
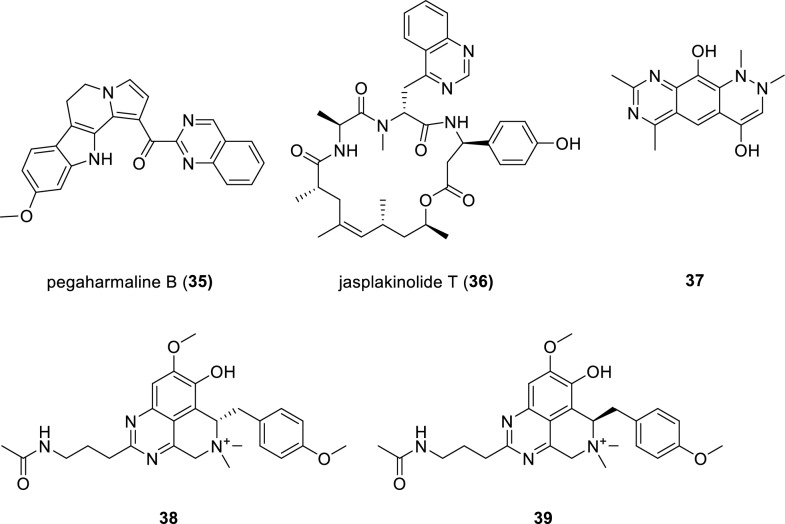
Chemical structures of compounds **35**–**39**

Investigation of the traditional medical herb *Peganum harmala* led to the isolation and identification of one quinazoline alkaloid pegaharmaline B (**35**) (Fig. 6). Notably, compound **35** represents a *β*-carboline-quinazoline hybrid with a highly conjugated C_24_ scaffold [54]. Compound **39** showed cytotoxic activity against HL-60 cell lines with an IC_50_ value of 16.6 μM. A family of unusual depsipeptide-polyketide compounds, known as jasplakinolide, has showed a significant cytotoxicity against renal, prostate and CNS tumor cell lines with good selectivity. These impressive data stimulate medicinal chemists to uncover more siblings in this family. Finally, jasplakinolide T (**36**), a new member characterized with quinazoline motif in this family, was isolated from marine sponges *Aulettas* sp. and *Jaspis splenedens*. The sponge materials from these two different species were combined together by the authors and subsequently subjected to isolation [55]. Unfortunately, the amount of isolated compound **36** was too limited to conduct a bioassay. An active metabolite **37**, possessing a quinazoline moiety, was isolated from the *Paenibacillus kribbensis.* The isolate **37** has strong broad-spectrum antifungal activity [56]. A pair of enantiomers **38** and **39** featuring quinazoline moieties, were isolated from the traditional Chinese medicine *Corydalis yanhusuo* and their absolute configurations were elucidated by electronic circular dichroism (ECD) calculations. Both compounds **38** and **39** showed acetylcholinesterase (AChE) inhibitory activity in a dose-dependent manner with the IC_50_ values of 14.07 and 13.75 μM, respectively [57].

### Pyrimidine-containing glycopeptides

Pyrimidine-containing glycopeptides, as defined in this review, refer to glycopeptides in which a pyrimidine moiety is incorporated into the molecular framework. Although these compounds include sugar residues, the sugar units are not directly linked to the pyrimidine core. Therefore, they are not classified as glycosylated pyrimidines in strict sense.

**Fig. 7:**
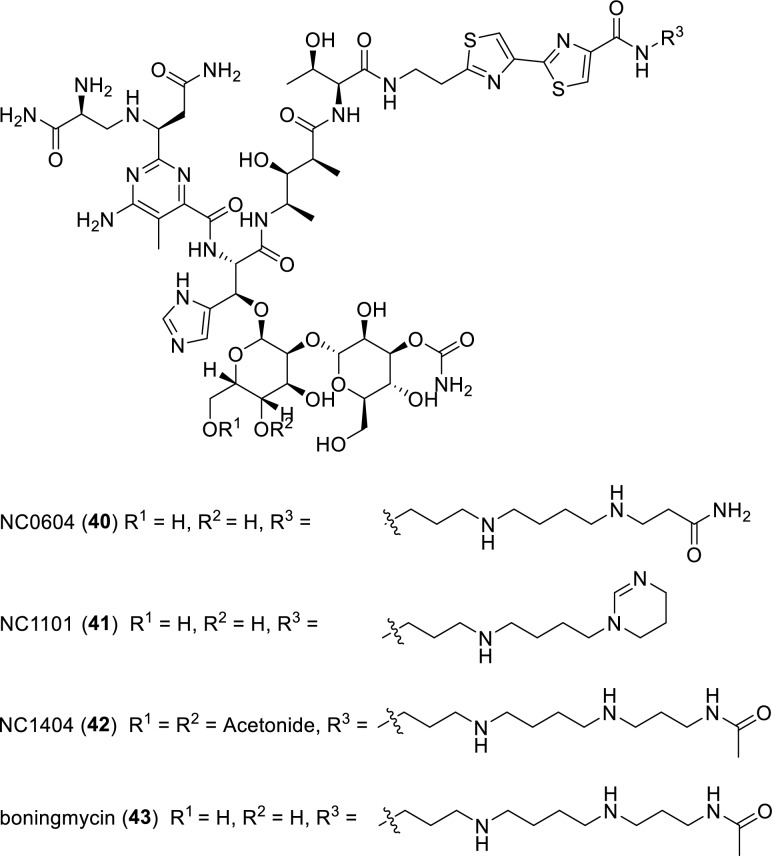
Chemical structures of compounds **40**–**43**

Four glycopeptide antibiotics, NC0604 (**40**), NC1101 (**41**), NC1404 (**42**) and boningmycin (**43**), which contain with tetrasubstituted pyrimidine moiety, are members of the bleomycin family (Fig. 7). Two representative members of this family, bleomycin A2 and bleomycin B2, mainly constitute commercial bleomycin, a clinically used anticancer drug [58]. Compounds **40–42** and **43** were isolated from the fermentation broths of *Streptomyces verticillus* var. *pingyangensis n. var* and *Streptomyces verticillus* var. *pingyangensis* n. sp., respectively [59–62]. Significantly, compared to bleomycin, compound **40** exhibited 3- to 9-fold greater antitumor activity than bleomycin against human HepG2, KB, MCF-7, HCT-116, BCG-813 and MCF-7 cell lines in vitro, while demonstrating substantially lower pulmonary toxicity. In parallel, compounds **41** and **42** also exhibited more potent inhibition against several tumor cell lines than bleomycin. Compound **43** demonstrated strong anticancer activity both in vitro and in vivo, mediated through the induction of apoptosis and cellular senescence, alongside with negligible pulmonary toxicity. A recent report reveals that PD-L1 upregulation in bleomycin-induced senescence requires activation of JAK/STAT signal pathway. These findings establish the groundwork for future studies aimed at elucidating the mechanisms of PD-L1 regulation during cellular senescence [63–65]. From a structural viewpoint, these four anticancer antibiotics **40–43** share the same core scaffold as clinical drug bleomycin, but feature distinct bithiazole tails that facilitate linkage with long-chain modifications. Therefore, further in-depth research on the structure–activity relationships (SAR), mode of action and toxicity profile of these four compounds is highly promising.

## Glycosylated pyrimidine derivatives

**Fig. 8:**
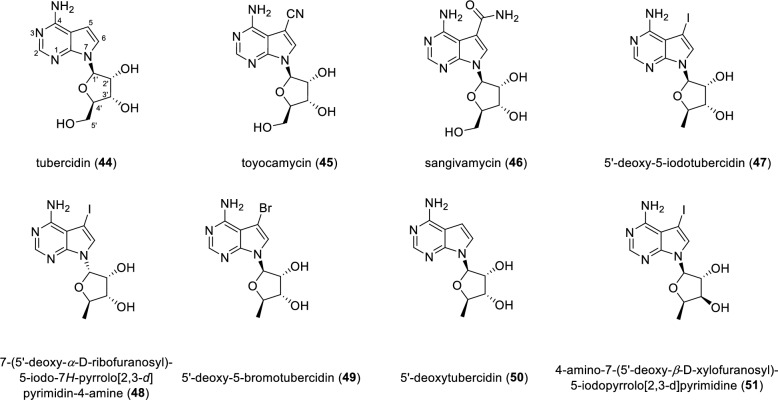
Chemical structures of compounds **44**–**51**

Glycosylated pyrrolopyrimidine derivatives constitute a subclass of natural products in which a sugar is directly linked to the pyrrolopyrimidine core. A *N*-glycosylated fused pyrimidine derivative (Fig. 8), tubercidin (**44**), was first isolated from *Streptomyces tubercidicus.* Tubercidin represents a distinctive class of pyrimidine derivatives featuring a pyrrolo[2,3-*d*]pyrimidine scaffold. This pyrrolopyrimidine nucleoside **44** exhibits broad biological activity, including notable antiviral and anticancer properties. Tubercidin has been shown to strongly inhibit the growth of *Streptococcus faecalis* (8043), with an IC_50_ value of 0.02 μM. Moreover, compound **44** displayed pronounced cytotoxicity against human cancer lines A549 and HST116, with IC_50_ values of 0.044 μM and 0.043 μM, respectively [66, 67]. In addition, compound **44** was recently identified as a potent methyltransferase 1(MTr1) inhibitor capable of suppressing influenza A replication [68]. Another tubercidin congener, toyocamycin (**45**), which bears a cyano group at C-5, was isolated from *Streptomyces toyocaensis*. Subsequent studies revealed that unamycin B, E-212 and vengicide isolated from *Streptomyces fungicidicus, Streptomyces* sp*.* E-212 and *Streptomyces vendargensis* respectively were structurally identical to toyocamycin [69, 70]. Compound **45** is a specific CDK9 inhibitor with an IC_50_ value of 79 nM. Compound **45** also inhibited XBP1 mRNA splicing with an IC_50_ value of 80 nM and suppressed tumor growth in a xenograft model of human multiple myeloma [71]. Sangivamycin (**46**) was first isolated from an unidentified *Streptomyces* species and later also rediscovered from the fermentation broth of *Streptomyces rimosus*. Compound **46** inhibited protein kinase C (PKC) with a *K*_i_ value of 10 μM and showed potent antiproliferative activity against HFF human fibroblasts, with an IC_50_ value of 0.08 μM [72, 73].

A pair of C1' anomers, 5'-deoxy-5-iodotubercidin (**47**) and 7-(5'-deoxy-*α*-D-ribofuranosyl)-5-iodo-7*H*-pyrrolo[2,3-*d*] pyrimidin-4-amine (**48**), were isolated from the red alga *Hypnea valendiae* [74]. Two pyrrolopyrimidine nucleosides, 5'-deoxy-5-bromotubercidin (**49**) and 5'-deoxytubercidin (**50**) were obtained as naturally occurring compounds from the ascidian *Didemnum voeltzkowi* [75]*.* Notably, these two tubercidin analogs (**49** and **50**) have previously prepared by semi-synthesized prior to their discovery as natural products [76]. Among them, compound **49** demonstrated potent inhibition of adenosine kinase with an IC_50_ value of 0.04 μM [77]. An unusual 5'-deoxyxylofuranosyl nucleoside, 4-amino-7-(5'-deoxy-*β*-D-xylofuranosyl)-5-iodopyrrolo[2,3-*d*]pyrimidine (**51**), was isolated from the ascidian *Diplosoma* sp. collected along the coast of Hateruma island, Okinawa [78]. Total synthesis of compound **51** was subsequently accomplished from the *D*-xylose, enabling unambiguous establishment of its absolute configuration [79].

## Purine-containing natural products

The heterocyclic aromatic organic compound purine consists of a pyrimidine ring fused to an imidazole ring. Purine derivatives constitute a wide variety of natural products containing pyrimidine ring system. Previous pioneering review covered by Rosemeyer provided a comprehensive summary about purine-containing natural products described before 2004 [28]. And several other reviews closely related to purine ring system highlight terpenyl-purine natural products and synthesis of natural purine nucleoside antibiotics [13, 14]. In this review section, natural products incorporating aromatic purine ring system will be exhaustively summarized.

### Purine alkaloids

Purine alkaloids constitute a diverse class of purine derivatives, encompassing *N*-alkylated purine alkaloids, terpenylated purines and miscellaneous metabolites characterized by an aromatic purine moiety. The* N*-alkylated subgroup includes compounds in which a purine nitrogen is substituted by alkyl chain. Terpenylated purines comprise conjugates in which the purine nucleus is fused or appended to sesquiterpenoid, diterpernoid, steroid and triterpenoid fragments. Compounds that do not fall into these categories are classified herein as miscellaneous purine derivatives. In addition, two terpenylated purine alkaloids (compounds **99** and **102**), in which a sugar unit is indirectly tethered to the purine core, are included in this section. In parallel, two purine alkaloids (compounds **101** and **103**) simultaneously bearing a sugar part and *N*-glycosylated purine are also compiled in this section, highlighting the importance of steroid and triterpenoid in this subgroup.

#### *N*-Alkylated purines

**Fig. 9:**
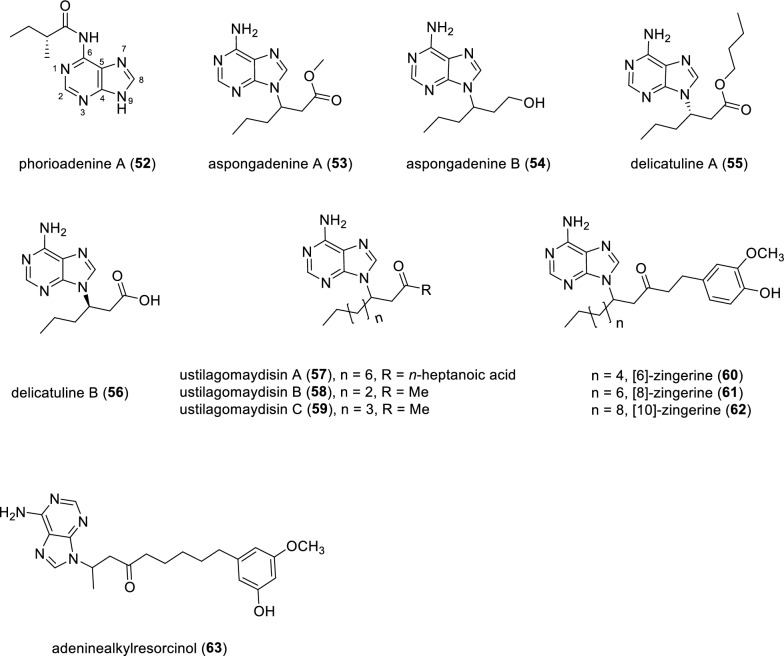
Chemical structures of compounds **52**–**63**

Chemical investigation of the southern Australian marine sponge *Phoriospongia* sp. (CMB-03107) afforded a naturally occurring alkaloid phorioadenine A (**52**), representing the first example of a 6-*N*-acyladeine (Fig. 9). The structure of compound **52** was elucidated by spectroscopic analysis and further confirmed by the chemical synthesis of racemic and enantiomeric forms of **52** [80]. Of note, *rac*-**52**, *ent*-**52** and three analogues (with differences located at the *N*-acyl side chains) were inactive toward the inhibition of larval development of *Haomonchus contortus*. Only compound **52** exhibited nematocidal activity against *H*. *contortus* with the LD_99_ value of 31 μg·mL^−1^, indicating the importance of the nature and chirality of the *N*-acyl side chain. Interestingly, the author conjured that compound **118** was likely to be a co-isolate, and subsequently completed the total synthesis of compound **118** (see Fig. 15). HPLC-ESIMS data analysis of authentic crude extract of *Phoriospongia* sp. (CMB-03107) successfully detected phorioadenosine A (**118**) as a minor co-existed secondary metabolite. It appears that the amount of compound **118** was too limited to be isolated and fully characterized.

Two adenine analogues, aspongadenines A and B (**53** and **54**), were naturally isolated as racemates from the insect *Aspongopus chinensis*. Chiral HPLC separation of these two racemic compounds **53** and **54** afforded two pairs of enantiomers, respectively. The absolute configurations of these optically pure compounds (*R*)-**53** and (*S*)-**53** were determined by calculated ECD. Subsequently, the absolute configurations of these optically pure compounds (*R*)-**54** and (*S*)-**54** were tentatively assigned by comparison of OR values with those of the enantiomers of compound **53** [81]. Two adenine-containing compounds delicatulines A and B (**55** and **56**) were isolated from the *n*-butanol extract of *Selaginella delicatula.* The absolute configurations of compounds **55** and **56** were determined by comparing their experimental ECD spectra with those of reported asponguanine A. Compounds **55** and **56** showed no inhibitory effect on HBV in the antiviral activity assay [82].

Ustilagomaydisins A–C (**57–59**), three adenine-derived alkaloids, were obtained from the pathogenic fungus *Ustilago maydis*. The OR values of the isolated compounds (**57–59**) were nearly zero, suggesting that these three isolates could be racemic. Compounds **57** showed weak activity to reverse the multidrug resistant in doxorubicin-resistant K562/A02 cell lines with verapamil as a positive control [83]. Phytochemical study on the methanol extract of ginger rhizomes (*Zingiber officinale*) led to the isolation and structure elucidation of three adenine derivatives [6]-,[8]-, and [10]-zingerines (**60**–**62**) as racemates. It’s noteworthy to point out that chemical structures of these three compounds (**60–62**) were further confirmed by chemical synthesis. A Michael addition between [6]-shogaol and adenine gave the desired molecule **60**. Using [8]-shogaol and [10]-shogaol under the identical reaction conditions, compounds **61** and **62** were smoothly prepared, respectively [84]. Adeninealkylresorcinol (**63**) is an alkylresorcinol derivative isolated from *Lasiodiplodia* sp., bearing an adenine moiety [85].

#### Terpenylated purines

**Fig. 10:**
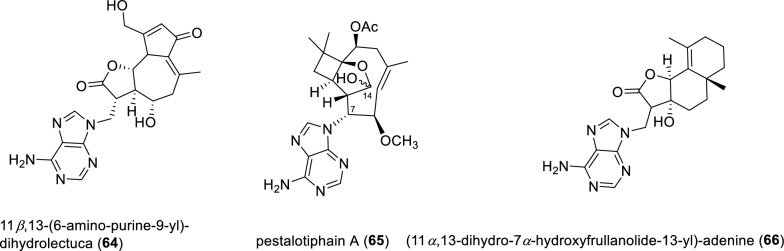
Chemical structures of compounds **64**–**66**

An adenine-11*β*,13-dihydrolectuca hybrid (Fig. 10), named 11*β*,13-(6-amino-purine-9-yl)-dihydrolectuca (**64**), was unlocked from *Lactuca tatarica*. Compound **64** displayed good inhibitory activity on Bruton’s tyrosine kinase with an IC_50_ value of 2.75 μM and also anti-inflammatory activitiy against NO release from mouse RAW264.7 macrophages induced by LPS with an IC_50_ value of 7.09 μM [86]. Pestalotiphain A (**65**), the first example of a *β*-caryophyllene derivative containing an adenine moiety, was identified from a plant-associate fungus *Pestalotiopsis hainanensis*. Compound **65** was originally described as a pair of hemiacetal epimers at C-14 (2.5:1 ratio in methanol-*d*^4^) based on a comprehensive analysis of NMR data. The absolute configuration of compound **65** was assigned by comparing its experimental ECD and OR value with those of the co-isolated pestalotiopsin C, a *β*-caryophyllene-type sesquiterpene with methoxy group at C-7 [87]. Aneudesmanolide-type sesquiterpenoid (**66**), combined with an adenine moiety, was characterized from the flower heads of *Sphaeranthus indicus.* In addition, semisynthesis of compound **66** was achieved through a Michael addition reaction of the natural isolate 7-hydroxyfrullanoide and adenine, further rationalizing its chemical structure [88].

**Fig. 11:**
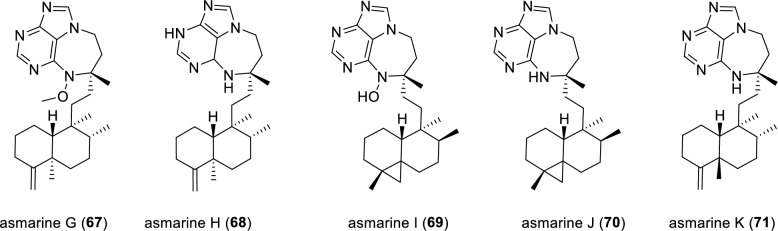
Chemical structures of compounds **67**–**71**

Five adenine-diterpene secondary metabolites, asmarines G–K (**67–71**), were isolated from the marine sponge *Raspailia* sp. (Fig. 11). All five isolated compounds (**67–71**) were evaluated for their in vitro cytotoxicities against a panel of human cancer cell lines. However, they were found to be inactive, in stark contrast to the potent congener asmarine A [89, 90].

**Fig. 12:**
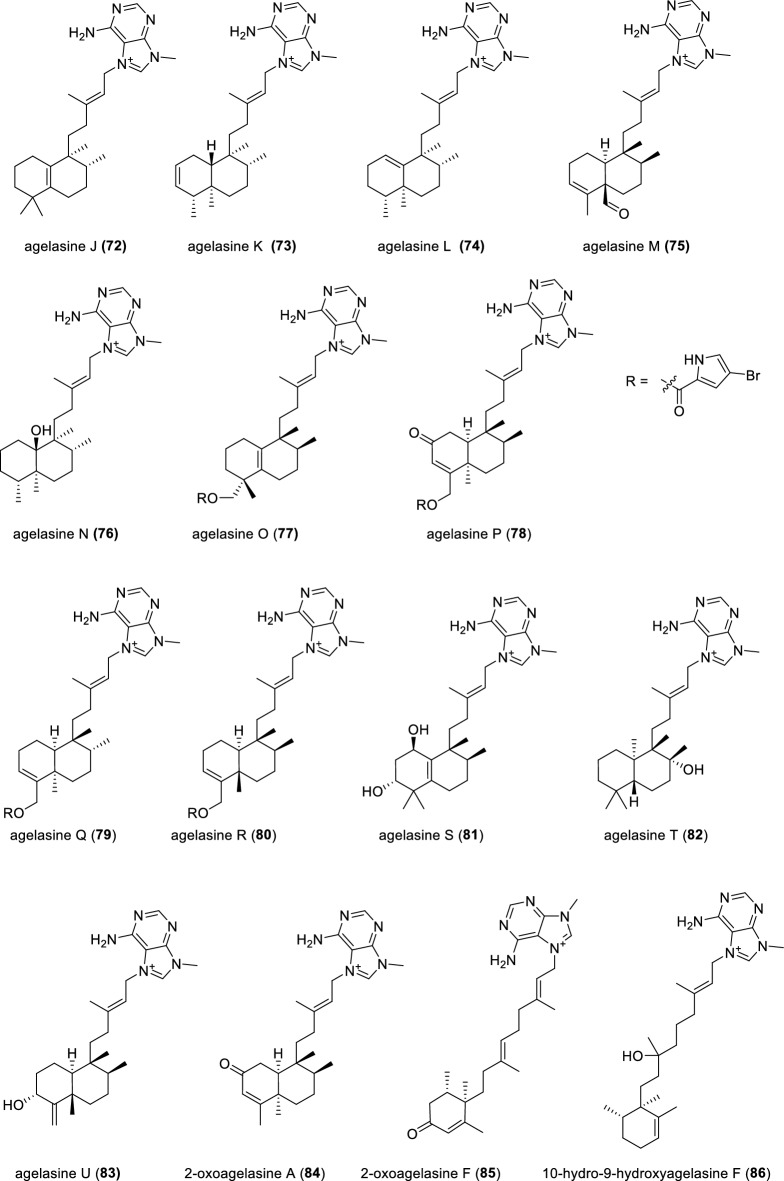
Chemical structures of compounds **72**–**86**

The genus *Agelas*, originating from the marine sponge, has been recognized as a rich source of diterpenes coupled with poplar functionality adenine [91]. Three diterpene alkaloids (Fig. 12), agelasines J–L (**72–74**), which belong to the agelasine family and possess a 9-*N*-methyladeninium, were isolated from the marine sponge *Agelas* sp*.* [92]*.* These three compounds (**72–74**) exhibited mild antimalarial activities against *Plasmodium falciparum* with IC_50_ values of 6.6, 8.3 and 18 μM, respectively. Another sibling agelasine M (**75**) were uncovered from the sponge *Agelas* sp. collected in Papua New Guinea [93]. A follow-up bioassay revealed that compound **75** showed notable antiparasitic activity against *Trypanosoma brucei* with an IC_50_ value of 3.0 μg·mL^−1^, alongside less cytotoxicity against Jurkat cell *T. brucei*.

The diterpene alkaloid agelasine N (**76**), was isolated from the Caribbean sponge *Agelas citrina*. The relative configuration of compound **76** was assigned by NOE correlation. However, the absolute configuration of compound **76** remains elusive due to the limited availability of the sample [94]. Agelasines O–U (**77–83**), possessing three different diterpene skeletons tethered with 9-*N*-methanyladenine motifs, were identified from the marine sponge *Agelas* sp. collected at Okinawa [95]. Conspicuous compounds (**77–80**) are endowed with the pyrrole esters and show antimicrobial activities against several bacteria and fungi. Three diterpene alkaloids containing an *N*-methyladenine moiety, 2-oxoagelasines A (**84**) and F (**85**) and 10-hydro-9-hydroxyagelasine F (**86**), were isolated from the Okinawan marine sponge *Agelas nakamurai*. Compound **84** inhibited the growth of *Mycobaterium smegmatis* with inhibition zones of 10 mm at 20 μg/disc [96].

**Fig. 13:**
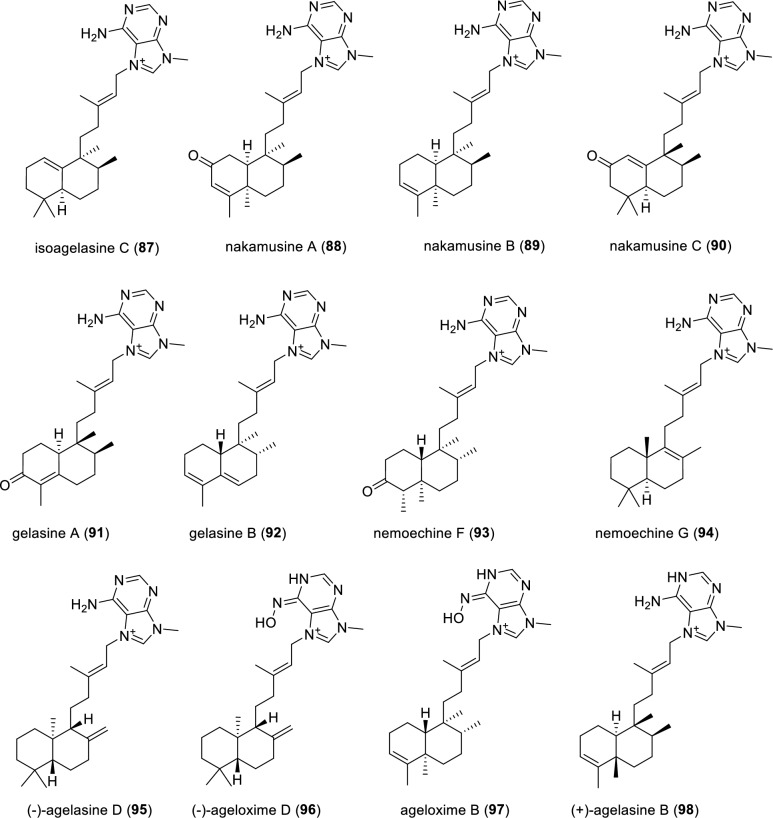
Chemical structures of compounds **87**–**98**

A congener of agelasine isoagelasine C (**87**), was isolated from the South China Sea sponge *Agelas nakamurai*, and its absolute configuration was deduced by ECD calculations (Fig. 13). Compound **87** displayed moderate antibacterial activities against *Proteusbacillus vulgaris* with MIC value of 18.75 μg·mL^−1^ [97]. Three adenine-containing alkaloids nakamusines A–C (**88**–**90**) were isolated from the Taiwanese sponge *Agelas nakamurai*. The absolute configuration of compound **88** was rationalized by ECD calculations [98]. Interestingly, co-occurring natural product 9-methyladenine was also isolated at the same time, indicating it might serve as a crucial biosynthetic building block to construct adenine-containing diterpene alkaloids in *Agelas* sponge. Two unique norditerpenoid alkaloids embedded with adenine moiety, gelasines A and B (**91** and **92**), were isolated from the marine sponge *Agelas*. sp. Compounds **91** and **92** were inactive in the antiparasitic assay against *T. brucei* [93]. Nemoechines F and G (**93** and **94**), featuring *N*-methyladenine-containing diterpene alkaloids, were isolated from the South China Sea sponge *Agelas* aff. *nemoechinata.* The absolute configuration of compound **93** was tentatively assigned on the basis of ECD calculations. Compound **94** showed weak activity against human lymphoblastic leukemia Jurkat cell lines with an IC_50_ value of 17.1 μmol·L^−1^. In the follow-up antimicrobial bioassay against *Staphyloccocus aureus* and *Bibrio parahemolyticus*, both compounds were inactive [99].

Two adenine-containing diterpene alkaloids (−)-agelasine D (**95**) and its oxime derivative (−)-ageloxime D (**96**) were isolated from the Indonesian sponge *Agelas nakamurai*. The ^1^H and ^13^C NMR data of compound **95** were identical to those of (+)-agelasine D, whose absolute configuration was established by chemical synthesis [100]. Interestingly, the experimental specific rotation value $[\alpha]_{\mathrm{D}}^{25}$ of compound **95** is −19 (*c* 0.5, MeOH) while the previously recorded specific rotation value $[\alpha]_{\mathrm{D}}^{25}$ of (+)-agelasine D is + 10.4 ((*c* 1.1, MeOH), suggesting that the compound **95** is the levorotatory enantiomer of ( +)-agelasine D. Compound **96** was also determined to be levorotatory with the similar method. Compounds **95** and **96** displayed cytotoxicity against L5178Y mouse lymphoma cell line with IC_50_ values of 4.03 and 12.5 μM, respectively. Furthermore, in an anti-fouling bioassay, both compounds **95** and **96** inhibited settling of larvae of *Balanus improvius* through their cytotoxicities. Ageloxime B (**97**) was isolated from the marine sponge *Agela mauritiana*. Compound **97** showed antifungal activity against *Cryptococcus neoformans*, antileishmanial activity against *Leishmania donovani* in vitro and antibacterial activity against *Staphylococcus aureus* and methicillin-resistant *Staphylococcus aureus* in vitro [101]. An ongoing exploration of the same sponge *Agela mauritiana* led to isolation of a diterpene alkaloid ( +)-agelasine B (**98**). The structure of compound **98** was determined as the dextrorotary isomer of agelasine B based on opposite optical rotation data for compound **98** ($[\alpha]_{\mathrm{D}}^{25}$ + 22.7 MeOH) and for (-)-agelasine B ($[\alpha]_{\mathrm{D}}^{25}$ -21.5, MeOH). Compound **98** not only showed moderate cytotoxicity against the cancer cell lines PC9, A549, HepG2, MCF-7 and U937 with IC_50_ values of ranging from 4.49 to 14.07 μM, but also displayed antibacterial activities against a panel of methicillin-resistant *Staphylococcus aureus* (MRSA) strains with MIC_50_ values of 1–2 μg·mL^−1^ [102].

**Fig. 14:**
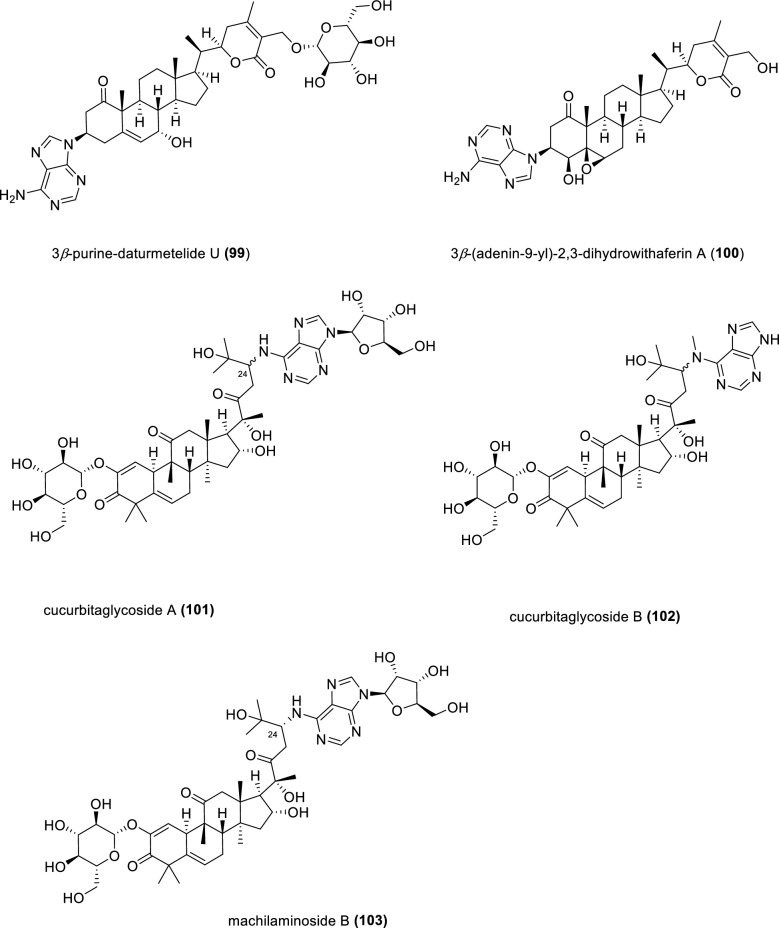
Chemical structures of compounds **99**–**103**

An adenine-containing compound 3*β*-purine**-**daturmetelide U (**99**) was isolated from the leaves of *Datura stramonium* (Fig. 14). Compound **99** showed excellent anti-inflammatory activity against BV2 microglia induced by lipopolysaccharides [103]. An unusual withanolide derivative 3*β*-(adenin-9-yl)-2,3-dihydrowithaferin A (**100**) possessing an adenine moiety was isolated from aeroponically grown *Withania somnifera,* an important herb known as Indian ginseng in Ayurvedic medicine [104, 105]. Cucurbitaglycosides A and B (**101** and **102**), the first cucurbitane triterpenoids tethered with a purine unit, were discovered from the fruits of *Cucurbita pepo* cv. *dayangua*. The relative configurations of compounds **101** and **102** at C-24 remain elusive. Compounds **101** and **102** displayed cytotoxic activity against the Hela cell line with IC_50_ values of 17.2 and 28.4 μg·mL^−1^ [106].

A unique cucurbitane triterpenoid coupled with an adenine motif, machilaminoside B (**103**), was isolated from the stem bark of *Machilus yaoshansis*. Despite high degree of structure similarity between compound **101** and compound **103**, confirming their identify as the same compound remains challenging. That is because they were obtained from different plant species, and their NMR data were recorded in different deuterated solvents. From another perspective, compounds **101** and **103** could be also classified as *N*-glycosylated nucleosides (*vide infra*). Compound **103** exhibited cytotoxic activities against five human cancer cell lines with IC_50_ values ranging from 0.3 to 0.8 μM without selectivity. Additionally, compound **103** also showed TNF-*α* secretion inhibitory activities in mouse peritoneal macrophages with an IC_50_ value of 0.1 μM [107].

#### Miscellaneous purine derivatives

**Fig.15:**
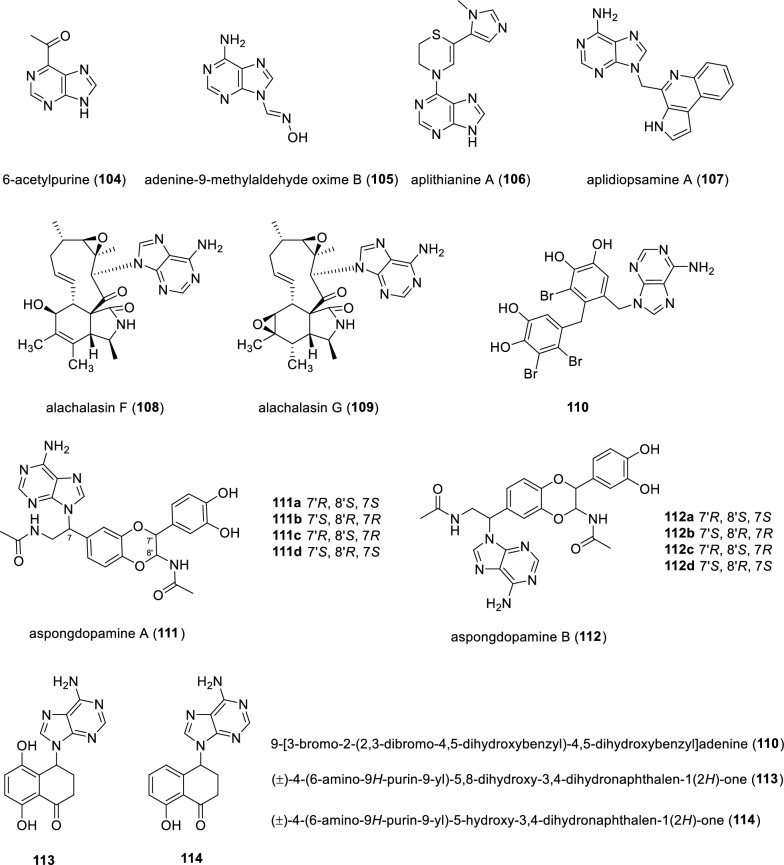
Chemical structures of compounds **104**–**112**

A purine derivative, 6-acetylpurine (**104**) was isolated from culture broth of *Cordyceps militaris* (Fig. 15). Notably, the OR value of compound **104** was −53.3 recorded in methanol while the compound **104** lacks an apparent chiral center [108]. However, no further explanation was provided by the author for this result. Adenine-9-methylaldehyde oxime B (**105**), an adenine-containing alkaloid with quite rare oxime unit [109], was discovered from the adult insect of *Allomyrina dichotoma*. Compound **105** showed inhibitory activities against the Gram-negative bacteria *Escherichia coli* with diameter of inhibition zone at 5.5 mm [110]. A high-throughput screen performed on approximately 326,000 prefractionated natural product mixtures generated by the National Cancer Institute’s (NCI) Program for Natural Product Discovery (NPNPD), leading to discovery a novel kinase inhibitor, namely, aplithianine A (**106**) isolated from the marine tunicate *Aplidium* sp. Unprecedent compound **106** showed potent inhibition against J-PKAc*α* and wild-type PKA with IC_50_ values of approximately 1 μM and 84 nM. Follow-up mechanistic studies indicated that compound **106** inhibited PKAc*α* catalytic activity by competitively binding to the ATP pocket. Additionally, concise total synthesis of compound **106** was conquered in 4 steps [19, 20].

Aplidiopsamine A (**107**), the first tricyclic alkaloid possessing the 3*H*-pyrrolo[2,3-*c*]quinoline linkage with an adenine moiety, was identified from the Australian ascidian *Aplidiopsis confluata*. Compound **107** demonstrated inhibition against two strains of *plasmodium falciparum*, including chloroquine sensitive (3D7) and resistant strains (Dd2) strains with IC_50_ values of 1.47 μM and 1.65 μM, respectively. More importantly, Compound **107** exhibited minimal toxicity toward human cell line HEK-293 [111, 112]. Alachalasins F and G (**108** and **109**) represent two unprecedented cytochalasins featuring adenine moieties, which were isolated from the fungus *Stachybotrys charatum.* Compound **108** showed weak antifungal activity against *Staphylococcus aureus* [113]*.* The isolation and characterization of a unique bromophenol derivative (**110**), coupled with an adenine moiety, from the red alga *Rhodomela confervoides* was described. Compound **110** represents the first example of bromophenols tethered with adenine moiety with the name 9-[3-bromo-2-(2,3-dibromo-4,5-dihydroxybenzyl)-4,5-dihydroxybenzyl]adenine [114].

Two insect-derived adducts aspongdopamines A and B (**111** and **112**) were identified from *Aspongopus chinensis,* bearing unprecedented hybrids consisting of *N*-acetyldopamine and adenine. Notably, both compounds **111** and **112** were naturally isolated as racemates. Separation via chiral HPLC afforded eight diasteroisomers **111a**-**111d** and **112a**-**112d**, respectively, due to the fixed *trans*-relationship of H-7' and H-8'. The absolute configurations of these stereoisomers were unambiguously determined by 14-steps total synthesis, ECD and VCD calculations [115]. Compounds **113** and **114,** namely**,** ( ±)-4-(6-amino-9*H*-purin-9-yl)-5,8-dihydroxy-3,4-dihydronaphthalen-1(2*H*)-one and ( ±)-4-(6-amino-9*H*-purin-9-yl)-5-hydroxy-3,4-dihydronaphthalen-1(2*H*)-one, respectively, possess the phenol-adenine hybrids originally isolated as scalemic mixtures from the flowers of *Juglans regia.* Separation of compound **113** and **114** via chiral HPLC afforded two pairs of enantiomers, respectively. Accordingly, the absolute configurations of these optically pure compounds (*R*)-**113** and (*S*)-**113** and (*R*)-**114** and (*S*)-**114** were determined by ECD data, respectively [116].

### *N*-glycosylated nucleosides

*N*-glycosylated nucleosides are defined as a class of molecular structures consisting of a base subunit adenine and a sugar subunit. Adenosine, a naturally occurring nucleoside, is one of the building blocks of DNR or RNA. In this section, *N*-glycosylated alkaloids with aromatic adenine moieties were recapitulated.

**Fig. 16:**
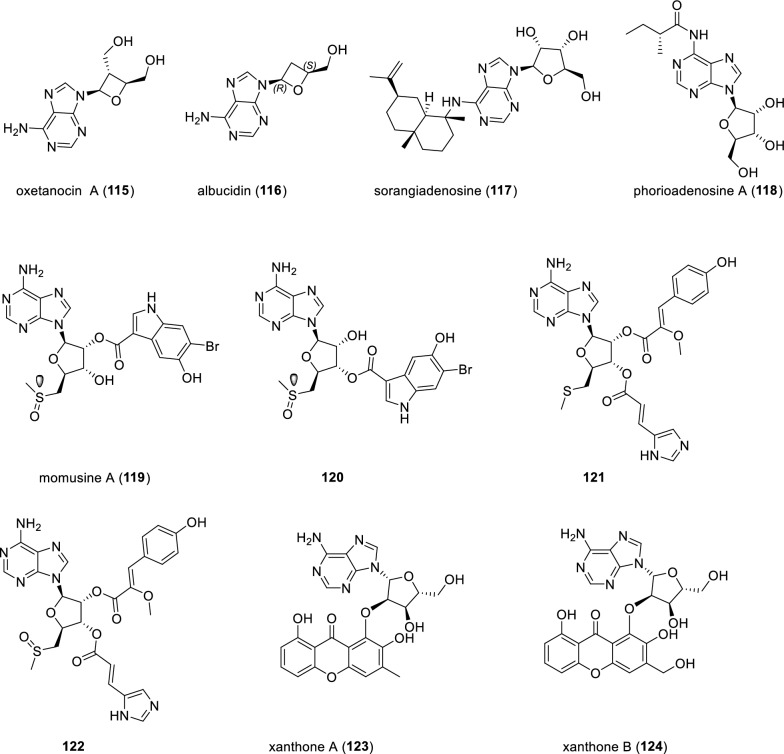
Chemical structures of compounds **115**–**124**

Oxetanocin A (**115**) represents the first naturally occurring alkaloid harboring an oxetanosyl-*N*-glycoside isolated from the culture broth of *Bacillus megaterium* NK84-0218 in 1986 (Fig. 16). Due to its interesting antiviral activity against human immunodeficiency virus (HIV), a series of oxetanocin A derivatives, including compound **116**, were obtained via chemical modification [117]. Nearly two decades later, compound **116** was described as a natural product isolated from the culture broth of *Streptomyces albus* subsp. *chlorinus* NRRL B-24108 via bioassay directed fractionation and was given the trivial name albucidin [118]. Compound **116** showed herbicidal activity against a broad spectrum of weeds. Recently, albucidin and its enantiomeric counterpart were synthesized, highlighting a late-stage reductive deiodination by visible light photocatalysis [119]. X-ray crystallography analysis of synthetic compounds pinpointed the absolute configuration of naturally occurring albucidin as 1*R*,3*S*. A very recent study has disclosed that two radical *S*-adenosyl-L-methionine (SAM) enzymes AlsB and AlsA are responsible for catalyzing a ring contraction step and eliminating a one-carbon fragment respectively, providing new insights into biosynthesis of albucidin [120]. Sorangiadenosine (**117**) possessing a glycosylated purine alkaloid with a bicyclic eudesmane-type sesquiterpene, was isolated from the culture broth of the myxobacterium *Sorangium cellulosum* collected from Korean soil. Compound** 117** showed moderate antibacterial activity against *Bacillus substilis* ATCC 6633 and *Micrococcus leuteus* IFC 12708 strains, with the MIC values of 6.25 and 6.25 μg·mL^−1^, respectively [121].

Chemical investigation of the *n*-BuOH-soluble fraction of the marine ascidian *Herdmania momus* led to the isolation and identification of two glycosylated purine derivatives momusine A (**119**) and its interconvertible transesterification isomer (**120**). Notably, compounds **119** and **120** afforded an equilibrium mixture of in a 56:44 ratio after HPLC separation upon standing at room temperature, indicating the presence of non-enzymatic acyl migration, as previously observed in similar precedents [122]. Furthermore, the presence of a chiral sulfoxide group in the compound **119** was ascribed to the co-isolated compound **120**. However, the sulfoxide configuration of compound **119** remains unknown. Compounds **121** and **122** are two complex nucleoside derivatives isolated from the ascidian *Atriolum robustum*. Structurally, compound **121** is quite similar to the compound **122**, with the striking differences being the methylthio group in compound **121** and the methylsulfinyl moiety in compound **122**. Compound **121** displayed affinity for A_1_ and A_3_ adenosine receptor with *K*_i_ values below 10 μM [123]. Xanthones A and B (**123** and **124**), two adenine-coupled xanthones, were identified from the endophytic ascomycete *Chalara* sp. 6661. And they were chemically induced by epigenetic manipulation of gene expression [124].

**Fig. 17:**
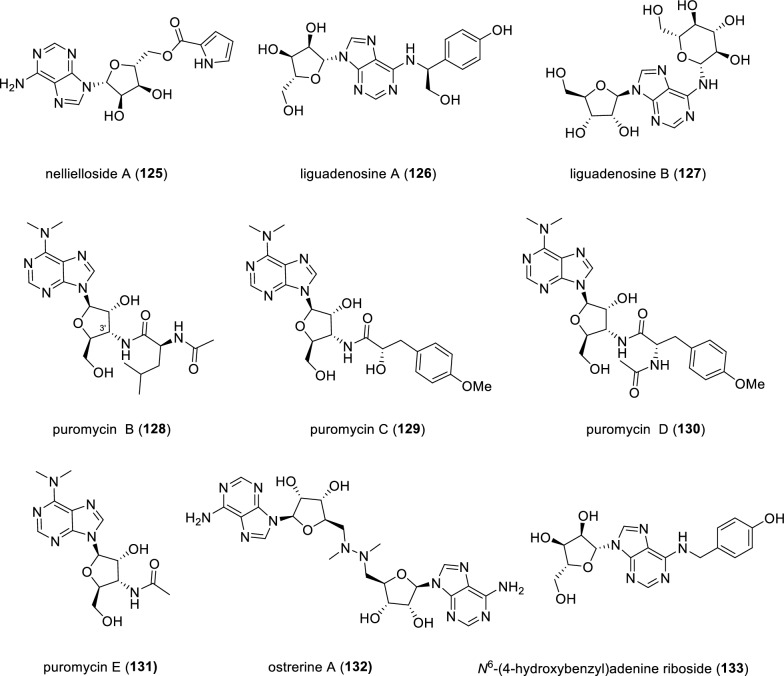
Chemical structures of compounds **125**–**133**

A glycosylated purine derivative, nellielloside A (**125**), was uncovered from the Pacific bryozoan *Nelliella nelliiformis* through NMR-guided isolation (Fig. 17). Compound **125** showed potent inhibitory activity against three kinases GSK3A, MAPK14, and RSK2 with IC_50_ values of 0.89, 1.00 and 0.80 μM, respectively [125]. Chemical investigation of the rhizomes of *Ligusticum striatum* DC. led to the isolation and characterization of two unusual *N*-10 substituted adenosine alkaloids, liguadenosines A and B (**126** and **127**) [126]. Although both compounds **126** and **127** showed significant anti-platelet aggregation activities in a concentration-dependent manner, their inhibitory effects were strikingly distinctive. Specifically, compound **126** displayed the strongest inhibitory effect at 10 μM and only weak inhibitory effect at 100 μM, whereas compound **127** showed the strongest inhibitory effect at 100 μM and weak inhibitory effect at 10 μM. The specific reason underlying this unusual phenomenon remains unclear. Four members of puromycin family, puromycins B–E (**128**–**131**), containing amino-nucleoside hybrids, were isolated from the Himalayan *Streptomyces* sp. PU-14-G collected at the Bara Gali region of northern Pakistan [127]. The absolute configuration of amino acid residue in compound **128** was determined to be L-leucine by Marfey’s method. Notably, compound **128** represents the first puromycin-related secondary metabolite with a 3'-*N*-amino acid substitution, in contrast to the classical 3'-*N*-tyrosinyl substitution in puromycin-type natural products. Ostrerine A (**132**), a dimeric alkaloid appended with a glycosylated purine, was isolated from the Quanzhou marine mollusk *Ostrea rivularis* [128]. A glycosylated purine, *N*^6^-(4-hydroxybenzyl)-adenine riboside (**133**), was obtained from the rhizomes of *Gastrodia elata*, a traditional Chinese medicine [129]. Compound **133** potently prevented PC12 cell apoptosis in a concentration-dependent manner with an IC_50_ value of 4.66 μM.

**Fig. 18:**
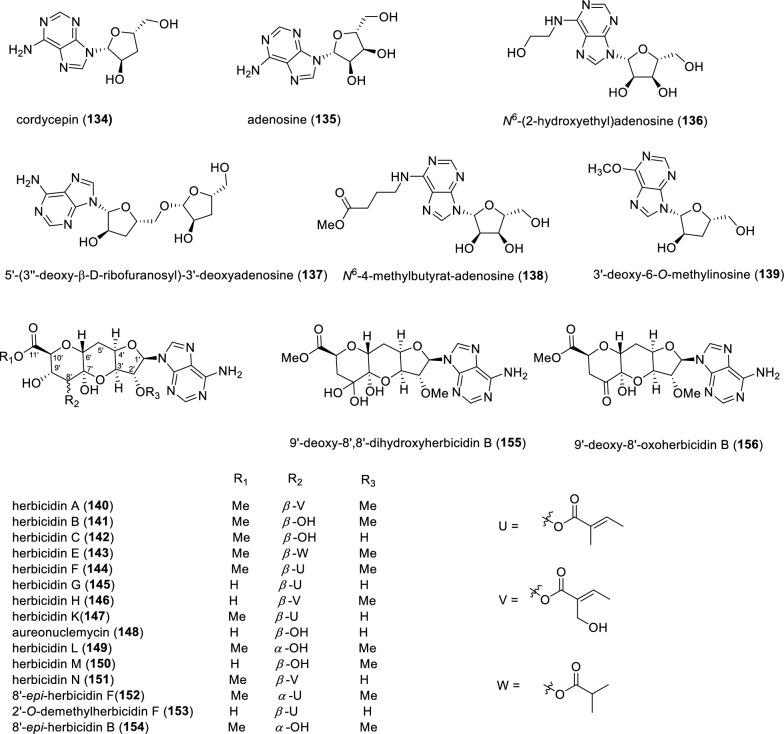
Chemical structures of compounds **134**–**156**

Four glycosylated purine derivatives (Fig. 18), cordycepin (**134**), adenosine (**135**), *N*^6^-(2-hydroxyethyl)adenosine (**136**) and 5'-(3''-deoxy-b-D-ribofuranosyl)-3'-deoxyadenosine (**137**), were identified from *Cordyceps militaris*, which is less well known to the public compared with *Cordyceps sinensis* (Dong Chong Xia Cao), a flagship species of the genus widely utilized in traditional medicine across Asian countries [130]. Vantagepoints of cordycepin chemistry continues to enjoy a renaissance, although it has been previously described long ago [131–134]. Another two glycosylated purine derivatives, *N*^6^-4-methylbutyrat-adenosine (**138**) and 3'-deoxy-6-*O*-methylinosine (**139**), were also disclosed from the fruiting bodies of *Cordyceps militaris* [135]. Herbicidins constitute a group of adenosine-derived nucleoside natural products, herbicidins A-C (**140**–**142**), herbicidins E–H (**143**–**146**), herbicidin K (**147**), aureonuclemycin (**148**), herbicidins L-N (**149**–**151**), 8'-*epi*-herbicidin F (**152**), 2'-*O*-demethylherbicidin F (**153**), 8'-*epi*-herbicidin B (**154**), 9'-deoxy-8',8'-dihydroxyherbicidin B (**155**) and 9'-deoxy-8'-oxoherbicidin B (**156**), which were isolated from various *Streptomyces* strains [136–139]. Structurally, they share a common scaffold decorated with an unusual tricyclic undecose, but differ in their substitution patterns at C2', C8', C9' and C11'-position. Aureonuclemycin (**148**), however, lacks substitution at these positions and consists solely of the nucleoside core. The characteristic tricyclic core of herbicidins comprises a furano-pyrano-pyran moiety appended with a (5-hydroxy)tiglyl unit [140–142]. The biosynthetic pathway was deciphered via feeding experiments, indicating that the tricyclic core originates from D-glucose and D-ribose and tiglyl unit is derived from an intermediate of L-isoleucine [143, 144].

## Discussion and outlook

The past two decades have witnessed a surge in effort to discover and characterize pyrimidine-containing natural products with fascinating structural attributes, such as ring-system complexity, richness in *sp*^3^-hybridized carbons, heteroatom contents and numerous stereocenters, providing an inspiration for medicinal chemists and chemical biologists to design novel molecules that leverage pyrimidine core as a privileged scaffold. Although the pyrimidine-containing natural occurrences in this review occupy only a limited proportion of total natural product repertoire identified hitherto, their structural diversity holds significant promise for anticancer, antiviral and antibacterial therapeutics. Despite being regarded as promising sources of novel biological activities, the full therapeutic value of these pyrimidine-containing natural products remains largely untapped due to the scarcity of authentic samples from natural sources. In this review, pyrimidine-containing compounds are structurally classified into different subgroups, offering a convenient reference for previously undescribed analogues in the foreseeable future and helping to intuitively renew interest in these underutilized compounds as promising synthetic targets.

Identified pyrimidine-containing natural products show diverse biological activities, including anticancer, antiviral and antibacterial activities. This interesting phenomenon closely mirrors the trends observed in synthetically derived small-molecule compounds. Several kinase inhibitors of pyrimidine-containing natural products, aplithianines A, nellielloside A and variolin B, exhibit potent activities in the nanomolar range and represent unique heterocyclic frameworks with considerable promise for the development of anticancer therapeutic. Tubercidin and their derivatives capable of suppressing the influenza hold potential as antiviral drug leads. However, the translation of these potent natural products into clinically viable drug candidates remains challenging. One promising strategy involves integrating these pyrimidine-containing natural products with rationally designed fragments to generate pseudonatural products, thereby enabling the systematic optimization of lead candidates. This approach may improve target selectivity, reduce cytotoxicity and minimize off-target effects. Moreover, further mechanism of action studies, particularly in animal-based disease models, would be highly beneficial for elucidating their target-specific structure–activity relationships and advancing these compounds towards therapeutic development.

With the advancement of new organic synthetic methodology developed, biosynthetic pathways elucidated and AI-driven cheminformatics integrated towards pyrimidine-containing compounds, daunting challenge posed by the paucity of authentic natural samples will be addressed, which will help unlock the full potential of these pyrimidine-containing compounds. Designing proteolysis-targeting chimaeras (PROTACs) based on pyrimidine-containing natural products offers an emerging option that holds potential to revolutionize contemporary drug discovery. Furthermore, continued efforts in the isolation and biological evaluation of such natural products will lay a solid foundation for future drug discovery.

## Data Availability

All the data and materials provided in the manuscript were obtained from references.
